# Supplementary material for: MetaRibo-Seq measures translation in microbiomes
Source: Nat Commun. 2020 Jun 29;11:3268. doi: 10.1038/s41467-020-17081-z (PMC7324362; doi:10.1038/s41467-020-17081-z)
Supplement: Supplementary file 10 — Supplementary Data 7 [file 41467_2020_17081_MOESM10_ESM.zip › File2/Confidence_High_Taxonomy/220653_out.krona.html]

Javascript must be enabled to view this page.

members
magnitude
magnitudeUnassigned
count
unassigned
taxon
rank

220653\_out

128

superkingdom
2
128

128
1239
phylum

species

SRS020869\_contig\_number\_contig-100\_259.193069
1263002
1

class
97
186801


SRS014235\_contig\_number\_7920
order
1
96
186802

216572
1
family

genus
459786
1


SRS150029\_contig\_number\_17597
1945593
1
species

species

SRS011084\_contig\_number\_28082SRS011134\_contig\_number\_contig-100\_190.190SRS011239\_contig\_number\_12405SRS011302\_contig\_number\_26343SRS012969\_contig\_number\_39617SRS013951\_contig\_number\_12777SRS013965\_contig\_number\_38272SRS015431\_contig\_number\_57301SRS015663\_contig\_number\_contig-100\_938.122086SRS015694\_contig\_number\_1340SRS016018\_contig\_number\_3230SRS016203\_contig\_number\_contig-100\_38834.38834SRS016335\_contig\_number\_contig-100\_3266.210087SRS017821\_contig\_number\_32857SRS018351\_contig\_number\_3872SRS018427\_contig\_number\_contig-100\_788.177823SRS018836\_contig\_number\_contig-100\_25770.74316SRS019068\_contig\_number\_93827SRS019496\_contig\_number\_11225SRS019968\_contig\_number\_contig-100\_12579.49904SRS020233\_contig\_number\_contig-100\_10840.178126SRS021948\_contig\_number\_25335SRS022071\_contig\_number\_36063SRS023526\_contig\_number\_18524SRS023914\_contig\_number\_16034SRS024132\_contig\_number\_40972SRS024435\_contig\_number\_17921SRS045826\_contig\_number\_16438SRS047433\_contig\_number\_contig-100\_33224.33225SRS048870\_contig\_number\_19683SRS049773\_contig\_number\_contig-100\_4991.158141SRS049959\_contig\_number\_32884SRS050752\_contig\_number\_13029SRS050925\_contig\_number\_contig-100\_6626.114938SRS051031\_contig\_number\_contig-100\_8823.143976SRS054352\_contig\_number\_15394SRS054905\_contig\_number\_17541SRS055017\_contig\_number\_25228SRS056273\_contig\_number\_22232SRS063190\_contig\_number\_13274SRS063985\_contig\_number\_21537SRS064276\_contig\_number\_31731SRS064757\_contig\_number\_4796SRS065397\_contig\_number\_contig-100\_24293.24294SRS074586\_contig\_number\_contig-100\_8656.8657SRS075078\_contig\_number\_2708SRS075341\_contig\_number\_3615SRS075821\_contig\_number\_14074SRS075984\_contig\_number\_23905SRS077024\_contig\_number\_4140SRS077194\_contig\_number\_contig-100\_7668.137717SRS077392\_contig\_number\_20161SRS078242\_contig\_number\_2716SRS078419\_contig\_number\_35728SRS098571\_contig\_number\_57318SRS098881\_contig\_number\_contig-100\_17990.109541SRS101376\_contig\_number\_20876SRS103987\_contig\_number\_contig-100\_9623.123707SRS1041032\_contig\_number\_14593SRS1041039\_contig\_number\_25390SRS1041092\_contig\_number\_8138SRS1041133\_contig\_number\_13763SRS1041137\_contig\_number\_1566SRS1041139\_contig\_number\_3802SRS1041157\_contig\_number\_990SRS104400\_contig\_number\_1733SRS140513\_contig\_number\_contig-100\_4033.42598SRS142503\_contig\_number\_56691SRS142542\_contig\_number\_2786SRS142923\_contig\_number\_14960SRS143417\_contig\_number\_17989SRS143466\_contig\_number\_21121SRS143523\_contig\_number\_6953SRS144183\_contig\_number\_38391SRS144506\_contig\_number\_47259SRS144537\_contig\_number\_926SRS144753\_contig\_number\_contig-100\_28256.73965SRS146812\_contig\_number\_38706SRS146813\_contig\_number\_contig-100\_6632.114288SRS147557\_contig\_number\_748SRS147614\_contig\_number\_10274SRS148196\_contig\_number\_38904SRS148424\_contig\_number\_4590SRS148817\_contig\_number\_13335SRS893172\_contig\_number\_15036SRS893187\_contig\_number\_contig-100\_1885.39278SRS893358\_contig\_number\_1587SRS893373\_contig\_number\_24228SRS893378\_contig\_number\_contig-100\_1450.43767SRS893383\_contig\_number\_35543SRS971275\_contig\_number\_43866SRS971276\_contig\_number\_33440
172733
92

species

SRS021484\_contig\_number\_18611
1897049
1

1
1897045

SRS077849\_contig\_number\_31230
species

species
1

SRS143895\_contig\_number\_36651
2044939


SRS1041031\_contig\_number\_16088
1263007
1
species

species
29
1263003

SRS012273\_contig\_number\_contig-100\_3924.204700SRS013098\_contig\_number\_46785SRS013521\_contig\_number\_contig-100\_708.73184SRS015960\_contig\_number\_contig-100\_2604.101018SRS017307\_contig\_number\_18009SRS019445\_contig\_number\_20073SRS022524\_contig\_number\_12065SRS042966\_contig\_number\_17151SRS043701\_contig\_number\_contig-100\_11731.86597SRS049896\_contig\_number\_contig-100\_5961.81778SRS049995\_contig\_number\_35616SRS052697\_contig\_number\_41325SRS053356\_contig\_number\_16007SRS058070\_contig\_number\_30264SRS063040\_contig\_number\_contig-100\_8346.204005SRS063518\_contig\_number\_contig-100\_13089.133707SRS065504\_contig\_number\_16873SRS075773\_contig\_number\_35146SRS076756\_contig\_number\_contig-100\_5081.80714SRS077231\_contig\_number\_9967SRS077641\_contig\_number\_contig-100\_12388.89271SRS078176\_contig\_number\_21285SRS098644\_contig\_number\_47473SRS1041141\_contig\_number\_13467SRS1041145\_contig\_number\_38797SRS104912\_contig\_number\_13136SRS147271\_contig\_number\_contig-100\_5561.184634SRS147346\_contig\_number\_contig-100\_229.199565SRS148253\_contig\_number\_contig-100\_1054.47079
